# Symptomatology and Serum Nuclear Magnetic Resonance Metabolomics; Do They Predict Endometriosis in Fertile Women Undergoing Laparoscopic Sterilisation? A Prospective Cross-sectional Study

**DOI:** 10.1007/s43032-021-00725-w

**Published:** 2021-09-15

**Authors:** Nicola Tempest, C. J. Hill, A. Whelan, A. De Silva, A. J. Drakeley, M. M. Phelan, D. K. Hapangama

**Affiliations:** 1 Department of Women’s and Children’s Health, Institute of Life Course and Medical Sciences, University Department, Liverpool Women’s Hospital, University of Liverpool, Member of Liverpool Health Partners, Crown Street, Liverpool, L8 7SS UK; 2 Liverpool Women’s Hospital NHS Foundation Trust, Member of Liverpool Health Partners, Liverpool, L8 7SS UK; 3 Hewitt Centre for Reproductive Medicine, Liverpool Women’s Hospital NHS Foundation Trust, Liverpool, L8 7SS UK; 4 Department of Obstetrics, Gynaecology and Women’s Health, University of Otago, 23A Mein Street, Newtown, Wellington, 6021 New Zealand; 5 HLS Technology Directorate, University of Liverpool, Liverpool, L69 3BX UK; 6 Department of Biochemistry and Systems Biology, Institute of Systems, Molecular and Integrative Biology, University of Liverpool, Liverpool, L69 7ZB UK

**Keywords:** Endometriosis, Incidence, Diagnosis, NMR, Metabolomics, Biomarkers

## Abstract

Endometriosis is a common, chronic inflammatory condition, thought to have a higher incidence in symptomatic women, yet, commonly associated symptoms do not always correlate with the presence or severity of disease and diagnosis requires surgery. We prospectively collected data and assessed symptomology and NMR spectroscopy-based metabolomics of 102 women undergoing laparoscopic sterilisation at a tertiary referral centre in a cross-sectional study. Twelve women were incidentally diagnosed with endometriosis (11.7%). According to the pre-operative questionnaire, presence and absence of many symptoms usually attributed to endometriosis were declared at similar frequencies in women with or without endometriosis. Women with endometriosis reported apparently more persistent heavy periods (50% vs 18.9%), prolonged periods (25% versus 7.8%) and problems conceiving (27.3% versus 9%) than those without endometriosis. NMR could not discern any distinguishable differences in the serum metabolome between those with and without endometriosis. Our paper highlights the complex symptomology experienced by women, regardless of a surgical diagnosis of endometriosis. Previous literature and the current study failed to identify clear, distinguishable symptoms or biomarkers pertinent to surgically confirmed endometriosis in the general population. Therefore, development of effective, non-invasive tests for identifying this heterogenous benign condition, endometriosis, is likely to be challenging.

**Supplementary Information:**

The online version contains supplementary material available at 10.1007/s43032-021-00725-w.

## Introduction

Endometriosis is an oestrogen-dependent chronic inflammatory condition affecting approximately 1 in 10 women of the general population and up to 1 in 2 with infertility [1, 2]. Women with endometriosis often suffer with dysmenorrhea as well as chronic pelvic pain and this can affect work, leisure, and social and intimate relationships [3]. Symptoms of pain, related to endometriosis, also affect the psychological health of women, compromising their quality of sleep and contributing to anxiety and depression [3–7]. The cost of endometriosis is considerable, both in terms of treatment and loss of productivity of the woman in society due to the disease [3], with pelvic pain and disease severity the major causes of work productivity loss [8].

The pathogenesis of endometriosis is poorly understood and diagnosis is through an invasive laparoscopy; thus, incidence data are usually from symptomatic women [2]. Despite the large number of studies conducted to date, non-invasive biomarker-based tests have not yet proven to be effective in diagnosing women with endometriosis in low-risk populations. There are no fully validated, symptom-based, patient-reported questionnaires for screening adult women for endometriosis [9]. A non-invasive test in this population will help to understand the natural history of endometriosis and also facilitate disease surveillance.

Metabolomics is the comprehensive analysis of small molecules in biological samples such as cells, tissues and biofluids. In humans, metabolomic profiling can be used to identify disease biomarkers and elucidate metabolic pathways involved in pathological processes [10]. Nuclear magnetic resonance (NMR) spectroscopy and mass spectrometry (MS) are the two most commonly employed techniques in metabolomics research [11, 12]. NMR metabolomic analysis provides highly reproducible, global quantitation of measurable analytes in biological samples in an unbiased and non-destructive fashion [13, 14]. For biomarker discovery, metabolomics has several advantages over transcriptomic and proteomic approaches. These include an overall reduction in data complexity due to the smaller number of metabolites compared to transcripts and proteins, the latter of which are further diversified by alternative splicing and post-translational modifications, respectively [15]. Furthermore, small molecule metabolites that relate to a disease phenotype are more likely to be secreted into systemic fluids than larger nucleic acids and proteins [15]. Such biofluids are ideal for biomarker identification and screening in low-risk populations because sample collection is simple and minimally invasive. Serum metabolites are attractive candidate biomarkers in endometriosis and previous studies have identified perturbations in the serum metabolome of women with symptomatic, minimal to mild stage disease [16, 17].

Symptoms associated with endometriosis, such as chronic pelvic pain, are not specific to women with endometriosis, and all women with surgically diagnosed endometriosis do not invariably report to have the classical endometriosis-associated symptoms. Therefore, one of the main challenges endometriosis researchers face is the lack of concordance between the disease and symptomatology, which in turn may have a diverse influence on different biomarker discovery studies. We thus hypothesised that by selecting a group of women who will incidentally undergo the diagnostic process irrelevant to the presence or absence of symptoms, and thus, we can capture obvious disease specific or pain-related symptoms and specific metabolomic aberrations. Women undergoing sterilisation in our cohort did not suffer from fertility-related problems and their presentation was to receive permanent, non-hormonal contraception. Our aim therefore was to identify if symptomology or serum metabolomics could differentiate between women with or without an incidental diagnosis of endometriosis in this population. We selected a group of women who attended hospital for the purpose of laparoscopic sterilisation and included only those without previous pelvic surgery or diagnosis of endometriosis to obtain a low-risk population to study the incidence of surgically diagnosed endometriosis and the potential associated symptomology and serum metabolomic signature. We anticipated a biomarker detected in a non-invasive test that can discern incidentally diagnosed endometriosis, particularly early stage disease, associated with minimal symptoms, will be of huge importance in future studies to understand the natural history and disease recurrence after optimal surgery, in the future.

## Methods

This study, was approved by the National Research Ethics Committee North West—Greater Manchester East (REC:13/LO/1247). All research was performed in accordance with relevant guidelines/regulations and in accordance with the Declaration of Helsinki.

### Patient Participation

Liverpool Women’s Hospital (LWH) has approximately 80,000 gynaecology consultations per annum and is a tertiary referral centre. After obtaining informed consent, this cross-sectional study was completed by prospectively and consecutively recruiting 102 eligible women from December 2013 to March 2017, in their reproductive years, with proven parity, undergoing laparoscopic sterilisation without a history of any previous pelvic surgery (total number of laparoscopic sterilisations during this time period was 317 (213 women excluded since they have had previous pelvic surgery and 2 women declined to take part)). Laparoscopic sterilisation is a form of non-reversible, permanent contraception available to women and the trained gynaecologist performing the procedure visualised the pelvis during this procedure and made a surgical diagnosis of endometriosis if present.

The women were asked to complete a questionnaire (Supplementary Figure I) pertaining to symptoms that could be attributed to endometriosis prior to laparoscopic sterilisation. The questionnaire included endometriosis-associated pain assessment questions, as per the BSGE pain questionnaire, additional demographic information and also self-reported diagnosis of other conditions that are often related to endometriosis such as overactive bladder syndrome and irritable bowel syndrome. All participants undertook a standard fasting period prior to surgery (> 6 h). During the laparoscopic sterilisation, the operating surgeon filled a pro forma documenting the presence or absence of endometriosis and the extent of the disease.

A sample of blood was collected from all of the women who underwent laparoscopic sterilisation immediately prior to induction of anaesthesia. These blood samples were subjected to NMR spectroscopic analysis to conclude if the serum metabolome could differentiate between women with and without endometriosis in this cohort.

### Sample Collection

Blood was collected into uncoated S-Monovette® Z-Gel tubes (Sarstedt, Leicester, UK) and allowed to clot for 20 min before centrifuging at 1,600 × *g* for 15 min at 4 °C. One-millilitre aliquots of serum were stored in sterile cryovials at − 80 °C.

### Sample Preparation for NMR

Aliquots were thawed and 125 µL of serum was diluted to a final volume containing 50% (v/v) serum, 40% (v/v) dd ^1^H_2_O (18.2 MΩ), 10% (v/v) 1 M PO_4_^3−^ pH 7.4 buffer (Na_2_HPO_4_, VWR International Ltd., Radnor, PA, USA and NaH_2_PO_4_, Sigma-Aldrich, Gillingham, UK) in deuterium oxide (^2^H_2_O, Sigma-Aldrich) and 1.2 mM sodium azide (NaN_3_, Sigma-Aldrich). Samples were vortexed for 1 min, centrifuged at 21,500 × *g* at 4 °C for 5 min and 200 µL transferred into 3-mm outer diameter NMR tubes using a glass pipette.

### Spectral Acquisition, Quality Control and Bucketing

Non-targeted 1D ^1^H NMR spectra were acquired at 37 °C using a 600 MHz Bruker Advance III spectrometer equipped with a TCI cryoprobe and chilled Sample-Jet autosampler. For each sample, a 1D ^1^H NMR standard experiment with the cpmgpr1d filters for selective observation of low molecular weight components with optimal water suppression was acquired; pulse sequence is vendor supplied using Carr-Purcell-Meiboom-Gill (CPMG) sequence (cpmgpr1d). Spectra were acquired with 32 transients a 30 ppm spectral width, 64 k points, 9.6 ms echo time and a 3.1 s acquisition time and a 4 s interscan delay. Full spectrum parameter sets are available with the data deposited at MetaboLights public repository (Haug et al. https://doi.org/10.1093/nar/gks1004).

Spectra were analysed to ensure conformity with the recommended minimum reporting standards [18, 19]. Spectra were aligned to a single formate peak at 8.46 ppm and all peaks within each spectrum were placed into buckets (160 buckets total).

### Metabolite Identification and Statistical Analysis

Metabolites were annotated using Chenomx NMR Suite 8.2 (332-mammalian metabolite library). Where possible the identities of the annotated metabolites were confirmed by comparison to in-house metabolite library in accordance to the Metabolites Standards Initiative best practice [18, 19]. Data is available with annotation via MetaboLights repository MTBLS2040.

Univariate and multivariate statistical analyses were conducted using MetaboAnalyst 4.0 [20]. Data sets were normalised to the sum of squares of all abundances (each sample equals 1) and Pareto scaled prior to analysis. For Student’s *t*-test analysis, *p* value false discovery rate (FDR) adjustment (Benjamini-Hochberg) was applied to compensate for multiple testing. The level of statistical significance was *p* values of < 0.05.

## Results

We included 102 women with no previous pelvic surgery aged 24–47 years (mean age 36 years) requesting permanent contraception who subsequently underwent a laparoscopic sterilisation in this study. The mean BMI of the patient cohort was 27.5. Of this population, 12 women were incidentally diagnosed with endometriosis (11.7%), out of which, 9 (8.8%) had stage I, and 3 (2.9%) had stage II disease.

The demographic features of women with and without surgically detected endometriosis were compared (Table 1). Irritable bowel syndrome (IBS) and overactive bladder symptoms were apparently more frequent in the group of women without endometriosis whilst women in the group diagnosed with endometriosis complained of a history of fertility problems more commonly (Table 1).

**Table 1:** Patient demographics

	Endometriosis (12)	No endometriosis (90)	Statistical significance*
Age in years, mean (Std Dev)	35 (5.4)	36.4 (5.6)	> 0.05
BMI, mean (Std Dev)	25.5 (3.1)	27.8 (5)	> 0.05
Smoker, *n* (%)	4/10 (40%)	35/78 (44.9%)	> 0.05
Current contraception			NA
Condoms	5 (41.7%)	17 (18.9%)	
Mirena coil	1 (8.3%)	7 (7.8%)	
Depo	3 (25%)	5 (5.6%)	
Implant	1 (8.3%)	4 (4.4%)	
COCP	1 (8.3%)	9 (10%)	
POP		13 (14.4%)	
Copper coil		7 (7.8%)	
Contraceptive patch		1 (1.1%)	
None	1 (8.3%)	18 (20%)	
Missing		4 (4.4%)	
NA		5 (5.6%)	
IBS, *n* (%)	0 (0%)	18 (20%)	> 0.05
Overactive bladder syndrome, *n* (%)	0/11 (0%)	3/89 (3.4%)	> 0.05
Sexually active, *n* (%)	11 (91.7%)	79/88 (89.8%)	> 0.05
Fertility problems	3/11 (27.3%)	8/89 (9%)	> 0.05

^*^Mann–Whitney *U* test

**Fig. 1:**
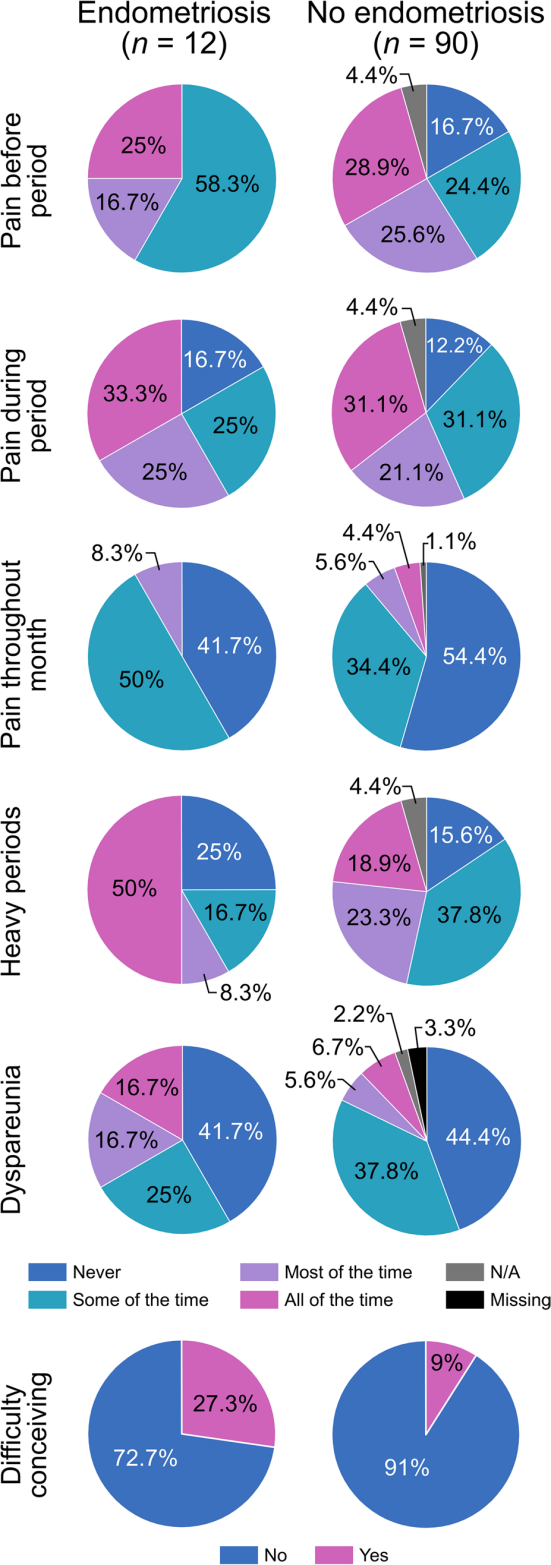
Selected symptomology of women with and without incidental diagnosis of endometriosis

When the patient-reported symptomatology was considered, most discerning symptoms that appeared to be preferentially associated with surgical detection of endometriosis were consistent pelvic pain throughout the month (8.3% vs 4.4%); persistent heavy (50% vs 18.9%) and prolonged periods (25% vs 7.8%); haematochezia with their periods (41.6% vs 12.2%); and persistent dyspareunia (33.4% vs 12.3%), which were all reported at higher rates by the women diagnosed with endometriosis as opposed to those who had no endometriosis at laparoscopy (Fig. 1, Table 2). Pain with exercise and activities of daily living (ADLs) was also reported at a more frequent rate by those diagnosed with endometriosis versus those without (16.7% vs 1.1% and 16.7% vs 4.4%) (Table 2). However, heterogeneity and limitation of these symptoms as diagnostic parameters are highlighted by the observation that a large proportion of women in the surgically diagnosed endometriosis group reported never having had heavy periods (25%) and prolonged periods (50%) (Fig. 1, Table 2).

**Table 2:** Preoperative symptoms as reported by questionnaire

	Endometriosis (12)	No endometriosis (90)
Pain before period		
Never		15 (16.7%)
Some of the time	7 (58.3%)	22 (24.4%)
Most of the time	2 (16.7%)	23 (25.6%)
All of the time	3 (25%)	26 (28.9%)
NA		4 (4.4%)
Pain during period		
Never	2 (16.7%)	11 (12.2%)
Some of the time	3 (25%)	28 (31.1%)
Most of the time	3 (25%)	19 (21.1%)
All of the time	4 (33.3%)	28 (31.1%)
NA		4 (4.4%)
Pain throughout month		
Never	5 (41.7%)	49 (54.4%)
Some of the time	6 (50%)	31 (34.4%)
Most of the time		5 (5.6%)
All of the time	1 (8.3%)	4 (4.4%)
NA		1 (1.1%)
Heavy periods		
Never	3 (25%)	14 (15.6%)
Some of the time	2 (16.7%)	34 (37.8%)
Most of the time	1 (8.3%)	21 (23.3%)
All of the time	6 (50%)	17 (18.9%)
NA		4 (4.4%)
Prolonged periods		
Never	6 (50%)	42 (46.7%)
Some of the time	2 (16.7%)	24 (26.7%)
Most of the time	1 (8.3%)	12 (13.3%)
All of the time	3 (25%)	7 (7.8%)
NA		4 (4.4%)
Missing		1 (1.1%)
Intermenstrual bleeding		
Never	6 (50%)	47 (52.2%)
Some of the time	6 (50%)	24 (26.7%)
Most of the time		8 (8.9%)
All of the time		7 (7.8%)
NA		4 (4.4%)
Pain with exercise		
Never	6 (50%)	40 (44.4%)
Some of the time	3 (25%)	32 (35.6%)
Most of the time	0	4 (4.4%)
All of the time	2 (16.7%)	1 (1.1%)
NA	1 (8.3%)	10 (11.1%)
Missing		3 (3.3%)
Pain with ADLs		
Never	5 (41.7%)	42 (46.7%)
Some of the time	5 (41.7%)	37 (41.1%)
Most of the time		6 (6.7%)
All of the time	2 (16.7%)	4 (4.4%)
Missing		1 (1.1%)
Fatigue		
Never	8 (66.7%)	37 (41.1%)
Some of the time	3 (25%)	29 (32.2%)
Most of the time		10 (11.1%)
All of the time	1 (8.3%)	12 (13.3%)
Missing		2 (2.2%)
Pain with bowel movements		
Never	7 (58.3%)	47 (52.2%)
Some of the time	4 (33.3%)	34 (37.8%)
Most of the time	1 (8.3%)	4 (4.4%)
All of the time		3 (3.3%)
Missing		2 (2.2%)
Tenesmus		
Never	9 (75%)	38 (42.2%)
Some of the time	3 (25%)	36 (40%)
Most of the time		9 (10%)
All of the time		4 (4.4%)
Missing		3 (3.3%)
Constipation		
Never	4 (33.3%)	30 (33.3%)
Some of the time	7 (58.3%)	45 (50%)
Most of the time	1 (8.3%)	10 (11.1%)
All of the time		4 (4.4%)
Missing		1 (1.1%)
Diarrhoea		
Never	4 (33.3%)	56 (62.2%)
Some of the time	8 (66.7%)	29 (32.2%)
Most of the time		4 (4.4%)
All of the time		
Missing		1 (1.1%)
Haematochezia with period		
Never	7 (58.3%)	78 (86.7%)
Some of the time	4 (33.3%)	11 (12.2%)
Most of the time	1 (8.3%)	
All of the time		
Missing		1 (1.1%)
Dysuria		
Never	9 (75%)	78 (86.7%)
Some of the time	3 (25%)	7 (7.8%)
Most of the time		3 (3.3%)
All of the time		
Missing		2 (2.2%)
Dyspareunia		
Never	5 (41.7%)	40 (44.4%)
Some of the time	3 (25%)	34 (37.8%)
Most of the time	2 (16.7%)	5 (5.6%)
All of the time	2 (16.7%)	6 (6.7%)
NA		2 (2.2%)
Missing		3 (3.3%)
Pain after intercourse		
Never	8 (66.7%)	58 (64.4%)
Some of the time	2 (16.7%)	21 (23.3%)
Most of the time	1 (8.3%)	5 (5.6%)
All of the time	1 (8.3%)	1 (1.1%)
NA		2 (2.2%)
Missing		3 (3.3%)
Avoided intercourse secondary to pain		
Never	8 (66.7%)	54 (60%)
Some of the time	3 (25%)	27 (30%)
Most of the time	1 (8.3%)	1 (1.1%)
All of the time		1 (1.1%)
NA		2 (2.2%)
Missing		5 (5.6%)

Some symptoms were similar in women with and without visual surgical detection of endometriosis, thus were less selective, and these included pain prior to their period (25% vs 28.9%) and during their period (33.3% vs 31.3%) (Fig. 1). Interestingly, never experiencing pre-menstrual pain was a symptom that appeared to be more prevalent in women without a surgical detection of endometriosis (16.7% vs 0%) (Fig. 1), thus representing a possible negative association with endometriosis. Persistent/frequent feeling of fatigue was reported by a higher proportion of women without endometriosis (24.4% vs 8.3%) (Table 2).

**Fig. 2:**
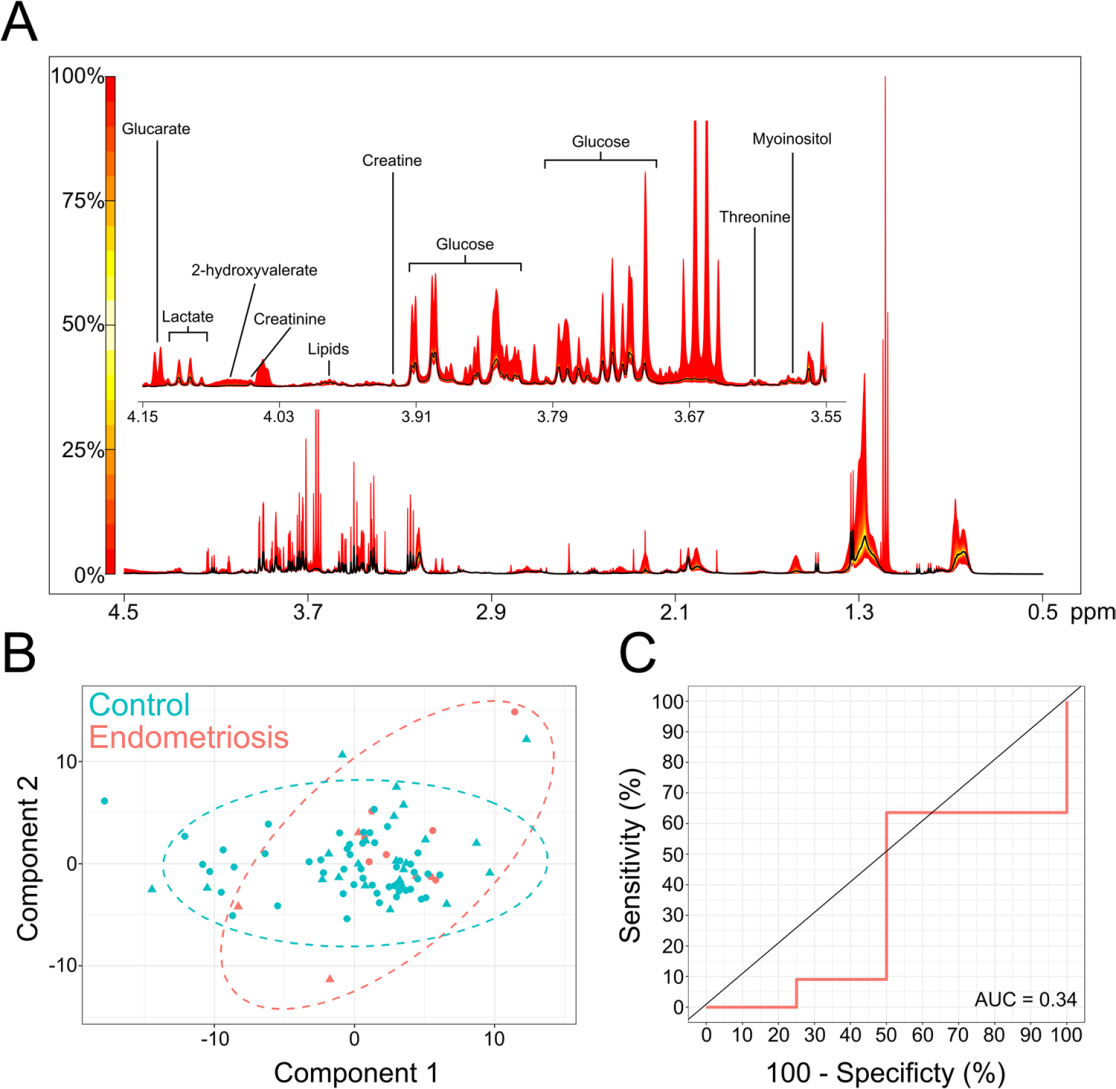
**A** Quantile plot of serum spectra (range 4.5–0.5 ppm) showing median plot (black line) and variation in all spectra (yellow to red) across the cohort. A detailed plot of the region 4.15–3.55 ppm is labelled with example metabolites to demonstrate peak annotation. **B** Multivariate PLS-DA scores plot of control and endometriosis serum samples demonstrating overlap between groups. Dashed lines represent 95% confidence regions. Training and test subsets are represented by circles and triangles, respectively. **C** ROC curve of the three-component PLS-DA model

NMR metabolomic analysis of serum samples was performed according to established protocols. Seventeen spectra (corresponding to samples from women without endometriosis) did not meet the recommended minimum reporting standards and were removed from subsequent analyses. In total, thirty-nine metabolites were annotated or identified in the serum samples according to the best practice recommendations of the metabolite standard initiative (MSI) (Supplementary Table 1) [18, 19]. A quantile plot of metabolite abundances demonstrated high variation in some spectral regions across the cohort (Fig. 2A). Univariate statistical analysis (false discovery rate adjusted) of all samples (12 with confirmed stage I or II endometriosis and 73 without endometriosis) did not identify any significantly different metabolites between groups (Table 3). Multivariate partial least squares discriminant analysis (PLS-DA) failed to discriminate between control and endometriosis metabolite profiles using a three-component model and leave-one-out cross validation (Fig. 2B). The area under the receiver operating characteristic (ROC) curve (AUC) of the three-component PLS-DA model confirmed that separation between groups was poor (AUC = 0.34; Fig. 2C). The sample cohort was refined to exclude smokers and those with a BMI > 30 (leaving 6 endometriosis and 25 control samples) and the analysis was repeated. PLS-DA model discrimination improved slightly (R_2_ 0.139 > 0.325), but was still unable to predict classification (Q_2_ negative, Table 3).

**Table 3:** NMR metabolomics statistical analysis

	Group	
	All: endometriosis (*n* = 12) vs no endometriosis (*n* = 73)	Refined: endometriosis (*n* = 6) vs no endometriosis (*n* = 25)
*t*-Test < 0.05 (FDR adjusted)	0	0
PLS-DA R_2_	0.139	0.325
PLS-DA Q_2_	− 0.306	− 0.505

## Discussion

In our cohort of 102 women undergoing laparoscopic sterilisation who had no previous pelvic surgery, 12 (11.7%) women were incidentally surgically diagnosed with endometriosis. The women diagnosed with endometriosis declared some typical symptoms associated with endometriosis apparently at higher frequency than those who did not have endometriosis at surgical examination of their pelvis, but none of the symptoms were exclusive to women with endometriosis. NMR analysis of the serum metabolome could also not discern any distinguishable differences between those with and without endometriosis.

Although previous authors have recorded incidental findings of endometriosis in women undergoing laparoscopic sterilisation ranging from 3 to 45.3% [21, 22], they have not explored the presence and types of endometriosis-associated symptoms in this group of women. For example, Tisso et al. described the prevalence of endometriosis to be 10% (36/360) in parous asymptomatic women, in agreement with our findings, but their limited assessment of associated pain mentions only that the prevalence of endometriosis was increased in patients with associated pain; 17.58% compared with 10.43% in women without pain (*p* = 0.06) [23]. This particular study, conducted over a 20-year period, does not detail the methodology for assessing symptomatology and thus is subject to bias due to recall, alteration in clinical/surgical practice, disease classification and assessment of types of endometriosis. In symptomatic women, previously reported incidence of endometriosis is higher, up to 70% [24]. In a recent cohort of young women with normal ultrasound findings and pelvic gynaecological examination, endometriosis was diagnosed in 20% [25]. The incidence of surgically diagnosed endometriosis in our patient cohort is less, similar to the background population rate of 10–15% [26]. Diagnosis of endometriosis requires laparoscopy, and the concordance of our data with previous estimates of prevalence in the general public validates our cohort as a low-risk group to model the general population, where available data is scarce. Furthermore, our collection of contemporaneous and prospective data, using pelvic pain questions from a validated British Society of Gynaecological Endoscopists (BSGE) questionnaire [27, 28], routinely used in clinical practice to assess endometriosis-associated symptoms, fills the existing gap in current literature.

Interestingly, a large proportion of women in our study complained of pelvic pain despite having endometriosis or not, whilst undergoing laparoscopy to achieve permanent contraception. Although these women were not attending the hospital seeking medical attention to alleviate their symptoms, the prevalence of pain and menstrual symptoms that we observed in this group highlights the high prevalence of these symptoms in the general public. For example, recent population data [29–32] demonstrated the high prevalence of dysmenorrhoea in women, which is reflected by the symptomatology of women in our study. The fact that many women endure the symptoms in the general population is important since the available evidence suggest that menstrual symptoms not only negatively impact the wellbeing of women and their productivity in society, but incur a huge cost to the economy [29, 30, 33–35].

In keeping with previous publications, a higher proportion of women with endometriosis in our patient cohort reported difficulty in conceiving [36]. Importantly, all women in our cohort had children and were undergoing an operation for permeant contraception, but 27.3% of the endometriosis group still gave a history of difficulty to conceive. This may correspond with the impact of minimal endometriosis on fertility as highlighted by previous authors [37]. Obstacles such as access to healthcare, availability of laparoscopy and acceptance of an operation as a mere diagnostic modality may prevent some symptomatic women from undergoing the procedure to obtain a diagnosis. Therefore, our cohort, who are usually not represented in the endometriosis biomarker studies, provide information on an unselected group of women for future researchers who aim to develop biomarkers suitable for assessing the natural history of endometriosis and disease surveillance.

In addition to assessing the surgical diagnosis and contemporaneous symptoms, we also examined serum biomarkers using NMR metabolomics. Interestingly, we did not identify clear discerning markers to differentiate those with a surgical diagnosis of endometriosis. Previous studies of the serum metabolome in endometriosis patients have identified significant changes in some metabolite profiles; NMR-based metabolomic analysis of serum from 22 women with laparoscopically confirmed endometriosis (stages I and II) identified discriminating metabolites including lactate, 3-hydroxybutyrate, amino acids, glycerophosphatidylcholine and glucose [16]. Another study identified 5 amino acids (alanine, leucine, lysine, proline and phenylalanine) as significantly altered in the serum of women with stage II endometriosis. Alanine alone could discriminate stage I endometriosis from healthy controls with 90% sensitivity [17]. Both studies were conducted exclusively in India, with no information provided on ethnic diversity. Dutta et al. [16] included 22 endometriosis patients, whilst Dutta et al. [17] included 20 stage I and 13 stage II patients. It is interesting to note that stages I and II endometriosis exhibited discriminant serum metabolomes, highlighting the heterogeneous pathology of endometriosis progression [17]. NMR signatures of the endometriosis metabolome have been derived from other biosamples including urine [38], follicular fluid [39] and endometrial tissue [17]. MS technologies have also been utilised to interrogate endometriosis-induced metabolite aberrations in peritoneal fluid [40], follicular fluid [39], plasma [41] and endometrial tissue [42]. One important consideration in any biomarker study is the specificity of the biomarker for the presence of the disease, the symptoms or both. We did not observe clear differences in the serum metabolome between women considering these groupings.

Blood samples included in our study were all taken just before general anaesthetic after > 6 h of fasting, thus were optimised for fasting glucose levels. Furthermore, our group of patients were within a relatively small age range and of single sex, thus were expected to demonstrate any striking differences pertinent to endometriosis. However, these samples were subject to metabolomic confounders including use of analgesics and contraceptives, patients at different phases of the menstrual cycle, obesity, diet, presence of pain (which may be due to potential undiagnosed pathological conditions) and smoking status. Indeed, BMI and smoking status have been shown to induce substantial confounding effects on the serum metabolome [43]. It is likely that these confounders, in addition to the relatively small sample size (only 12 women with surgically detected mild endometriosis), may have contributed to our data not demonstrating discernible differences between the metabolite profiles for women with and without endometriosis. The exclusion of smokers and those with high BMI from the multivariate analyses did not improve the predictive power of the models, potentially due to a further reduction in the endometriosis group sample size. The women who were diagnosed with endometriosis only had relatively mild disease (stages I and II). Although these women reported symptoms, the fact that they have not sought medical help may imply that in addition to the low disease volume/burden, their symptoms and causative metabolomic aberrations are minimal, and thus, we were unable to detect an obvious difference. Further investigations are needed in the future to examine the metabolomic differences with a sufficiently larger sample size that would allow normalising to all possible confounders. However, our data on symptomatology and serum metabolomics in a population of women undergoing laparoscopic sterilisation informs the research community of the challenges relevant to the development of efficient diagnostic tests for this heterogeneous condition, endometriosis, where the surgically assessed disease volume and severity do not necessarily correlate with symptomatology. The lack of unique symptoms or metabolomic markers in our study and in a plethora of previous studies certainly cast doubts on the feasibility of developing a single, non-invasive diagnostic test that is likely to be effective in general population-based screening.

## Supplementary Information

Below is the link to the electronic supplementary material.

Supplementary file1 (DOC 56 KB)

Supplementary file2 (DOCX 16 KB)

## Data Availability

NMR metabolomics data is deposited and publicly available from the MetaboLights database (MTBLS2040).
