# Supplementary material for: Symptomatology and Serum Nuclear Magnetic Resonance Metabolomics; Do They Predict Endometriosis in Fertile Women Undergoing Laparoscopic Sterilisation? A Prospective Cross-sectional Study
Source: Reprod Sci. 2021 Sep 15;28(12):3480–90. doi: 10.1007/s43032-021-00725-w (PMC8580895; doi:10.1007/s43032-021-00725-w)
Supplement: Supplementary file 1 — Supplementary file1 (DOC 56 KB) [file 43032_2021_725_MOESM1_ESM.doc]

**Incidence of Endometriosis Study**

**Patient Questionnaire**

General lifestyle

1. Do you have lower backache?

Never / some of the time / most of the time / all of the time

1. Do you have pain during exercise?

Never / some of the time / most of the time / all of the time / NA

1. Do you have pain with day to day activities (eg –housework, recreational activities)

Never / some of the time / most of the time / all of the time

1. Do you suffer from fatigue or excessive tiredness?

Never / some of the time / most of the time / all of the time

Menstrual function

1. Do you have pain before the start of a period?

Never / some of the time / most of the time / all of the time / NA

1. Do you have pain during a period?

Never / some of the time / most of the time / all of the time / NA

1. Do you have pain throughout the month not related to a period?

Never / some of the time / most of the time / all the time

1. Do you have heavy periods?

Never / some of the time / most of the time /all of the time/ NA

1. Do you have prolonged periods (more than 7 days)?

Never / some of the time / most of the time / all of the time/ NA

1. Do you have bleeding or spotting in-between periods?

Never / some of the time / most of the time / all of the time/ NA

Bowel Function

1. How many times do you open your bowels during the day?
2. How many times do you open your bowels at night?
3. Do you have pain with bowel movements?

Never / some of the time / most of the time / all of the time

1. Do you have sensation of incomplete emptying of your bowels?

Never / some of the time / most of the time / all of the time

1. Do you suffer from constipation?

Never / some of the time / most of the time / all of the time

1. Do you suffer from diarrhoea?

Never / some of the time / most of the time/ all of the time

1. Have you had bleeding from your back passage at the same time as your period?

Never / some of the time / most of the time / all of the time

1. Have you been diagnosed with irritable bowel syndrome?

Yes / No

Bladder function

1. How many times during the day do you pass urine?
2. How many times during the night do you pass urine?

5. Do you have pain when passing urine?

Never / some of the time / most of the time / all of the time

1. Have you been diagnosed with urinary urgency (overactive bladder syndrome)?

Yes / No

Sexual function

1. Are you sexually active?

Yes / No

1. Do you have pain with deep penetration during sexual intercourse?

Never / some of the time / most of the time / all of the time / NA

1. Do you have pain after sexual intercourse?

Never / some of the time / most of the time / all of the time / NA

1. Have you avoided sexual intercourse as a result of pain?

Never / some of the time / most of the time / all of the time/ NA

Contraceptive History

1. Have you ever taken the combine oral contraceptive pill?

Yes / No If yes for how long?

1. Have you ever taken progestegen only (mini pill)?

Yes / No If yes for how long?

1. Have you ever taken the depoprovera injection?

Yes/ No If yes for how long?

1. Have you ever used the contraceptive implant (Implanon/ Norplant)? Yes/ No If yes for how long?
2. Have you ever used the contraceptive coil (copper non-hormonal)? Yes/ No If yes for how long?
3. Have you ever used the mirena coil?

Yes/ No If yes for how long?

1. What is your current method of contraception?
2. Have you breastfed your children?

Yes/ No If yes, how long did you fully breast feed for?

Conception

1. Did you ever have any difficulties conceiving?

Yes / No

1. If yes, did you require treatment for subfertility?
2. If yes please provide details on the treatment received -
