# Supplementary material for: Symptomatology and Serum Nuclear Magnetic Resonance Metabolomics; Do They Predict Endometriosis in Fertile Women Undergoing Laparoscopic Sterilisation? A Prospective Cross-sectional Study
Source: Reprod Sci. 2021 Sep 15;28(12):3480–90. doi: 10.1007/s43032-021-00725-w (PMC8580895; doi:10.1007/s43032-021-00725-w)
Supplement: Supplementary file 2 — Supplementary file2 (DOCX 16 KB) [file 43032_2021_725_MOESM2_ESM.docx]

| **Database identifier** | **Metabolite identification** | **Reliability** |
| --- | --- | --- |
| HMDB0000008 | 2-hydroxybutyrate | MSI level 2 |
| HMDB0000407 | 2-hydroxyisovalerate | MSI level 2 |
| HMDB0001863 | 2-hydroxyvalerate | MSI level 2 |
| HMDB0000357 | 3-hydroxybutyrate | MSI level 2 |
| HMDB0000042 | Acetate | MSI level 1 |
| HMDB0000060 | Acetoacetate | MSI level 1 |
| HMDB0000097 | Choline | MSI level 1 |
| HMDB0000094 | Citrate | MSI level 1 |
| HMDB0000064 | Creatine | MSI level 1 |
| HMDB0000562 | Creatinine | MSI level 1 |
| HMDB0002199 | Desaminotyrosine | MSI level 2 |
| HMDB0000122 | D-glucose | MSI level 1 |
| HMDB0000663 | Glucarate | MSI level 2 |
| HMDB0000721 | Glycylproline | MSI level 2 |
| HMDB0001525 | Imidazole | MSI level 1 |
| HMDB0000863 | Isopropanol | MSI level 2 |
| HMDB0000190 | Lactate | MSI level 1 |
| HMDB0000161 | L-alanine | MSI level 1 |
| HMDB0000517 | L-arginine | MSI level 1 |
| HMDB0000148 | L-glutamate | MSI level 1 |
| HMDB0000641 | L-glutamine | MSI level 1 |
| HMDB0000177 | L-histidine | MSI level 1 |
| HMDB0000172 | L-isoleucine | MSI level 1 |
| HMDB0000687 | L-leucine | MSI level 1 |
| HMDB0000182 | L-lysine | MSI level 1 |
| HMDB0000159 | L-phenylalanine | MSI level 1 |
| HMDB0000162 | L-Proline | MSI level 1 |
| HMDB0000167 | L-threonine | MSI level 1 |
| HMDB0000158 | L-tyrosine | MSI level 1 |
| HMDB0000883 | L-valine | MSI level 1 |
| N/A | Lipid | MS level 2 |
| HMDB0000169 | Mannose | MSI level 2 |
| N/A | Mobile lipids | MSI level 3 |
| N/A | Mobile lipids HDL | MSI level 3 |
| N/A | Mobile lipids LDL | MSI level 3 |
| N/A | Mobile lipids VLDL | MSI level 3 |
| HMDB0000211 | myo-Inositol | MSI level 1 |
| HMDB0031419 | N-nitrosodimethylamine | MSI level 2 |
| HMDB00001881 | Propylene-glycol | MSI level 1 |

**Supplementary Table 1.** Metabolites annotated in serum samples using Chenomx (level 2/3) or identified using in-house standards (level 1) as recommended by Metabolomics Standard Initiative (MSI) [12, 13].
